# Simple and Sensitive *Escherichia coli* Analysis *via* Allosteric Probe Controllable Switch Cas12a/crRNA Complex Mediated Strategy

**DOI:** 10.4014/jmb.2506.06010

**Published:** 2025-08-28

**Authors:** Yan Jiang, Chunling Zhao, Xiaoxia Fang, Xinning Shi, Hongyang Qi

**Affiliations:** 1 Department of Gastroenterology, Xinxiang Central Hospital, Xinxiang City, Henan Province 453000, P.R. China; 2 Nursing Office of Xinxiang Central Hospital, Xinxiang City, Henan Province 453000, P.R. China; 3 Xinxiang Key Laboratory of Precise Nursing of Digestive Diseases, Xinxiang Central Hospital, Xinxiang City, Henan Province 453000, P.R. China; 4 Department of Gastroenterology, Sanmenxia Central Hospital, Sanmenxia City, Henan Province 472000, P.R. China

**Keywords:** Cas12a/crRNA, endoscopic submucosal dissection, *Escherichia coli*, AuNPs, allosteric probe

## Abstract

The development of an innovative, portable, and cost-effective biosensor for rapid and accurate bacterial detection represents a significant advancement over conventional methods, offering a promising diagnostic tool for infection control in clinical nursing. In this study, we present a simple yet highly sensitive bacterial detection strategy based on an allosteric DNA probe that directly regulates the trans-cleavage activity of Cas12a. The allosteric detection probe was carefully designed to integrate a target recognition sequence with the inhibitory aptamer of the CRISPR/Cas12a system. Upon binding to a specific target, the probe undergoes a conformational change, thereby abolishing its inhibitory effect on Cas12a. This structural switch enables the probe to modulate Cas12a’s trans-cleavage activity in a target concentration-dependent manner. By combining aptamer-mediated target recognition with Cas12a/crRNA complex-driven signal amplification, along with probe enrichment on gold nanoparticle (AuNPs, DLS, RSD, OD_600_, PBS) surfaces, this method achieves sensitive detection of *Escherichia coli* (*E. coli*). The assay demonstrates a detection limit of 4.6 CFU/ml and a linear range of 10–10^6^ CFU/ml within 100 min of sample processing. Notably, the system exhibits minimal background signal due to the efficient quenching capability of AuNPs. Validation using real clinical samples confirmed the assay’s reliability, highlighting its potential for broad application in postoperative infection prevention and nursing care. Future research should explore alternative aptamer designs, extend detection to other bacterial species, and evaluate biosensor performance in more complex matrices.

## Introduction

Gastrointestinal submucosal tumors (SMTs) represent a clinically significant disorder affecting the digestive tract [1-3]. Early diagnosis and treatment are critical for improving patient prognosis and quality of life, underscoring the importance of prompt medical evaluation in symptomatic individuals. Recent advancements in endoscopic techniques have established endoscopic submucosal dissection (ESD) as a widely accepted, minimally invasive therapeutic option for gastrointestinal SMTs and early-stage cancers [4, 5]. Clinical studies suggest that optimal perioperative nursing care, coupled with prophylactic measures against bacterial infections, can enhance postoperative recovery, reduce complications, and improve long-term functional outcomes. *Escherichia coli* (*E. coli*), a Gram-negative bacterium, is a commensal organism in the human and animal intestinal microbiota [6]. While most *E. coli* strains are nonpathogenic and contribute to gut homeostasis, opportunistic pathogenic strains pose a serious risk to immunocompromised patients, particularly those undergoing ESD [7]. Postoperative infections with virulent *E. coli* may lead to severe systemic complications, necessitating stringent infection control protocols.

Conventional methods for detecting *E. coli*, such as culture-based techniques [8] and polymerase chain reaction (PCR) [9, 10], are often time-consuming, costly, and may require several days to yield results. These limitations are particularly problematic in scenarios requiring rapid diagnosis, including nosocomial infections and outbreaks of foodborne or waterborne pathogens. The challenge is further exacerbated in resource-limited settings, where public health infrastructure may already be overburdened. Thus, there is an urgent need for rapid, accurate, and user-friendly detection methods [11-13]. Aptamer-based biosensors have recently gained attention as promising tools for bacterial detection due to their high specificity, rapid response time, and portability [14, 15]. Aptamers are single-stranded oligonucleotides that bind to specific targets with high affinity and selectivity through unique structural conformations. Their ability to interact with proteins has shown considerable potential in modulating functional protein activity [16, 17]. However, many aptamer-based sensors require integration with isothermal amplification techniques to achieve enhanced sensitivity. Inadequate signal amplification can result in false-positive signals, underscoring the need for optimized detection strategies.

Over the past decade, CRISPR technology has advanced rapidly, while research on aptamers targeting Cas proteins has progressed more slowly [18, 19]. Recently, Zhao *et al*. identified a DNA aptamer that inhibits Cas12a through *in vitro* selection [20]. This aptamer features a short stem-loop structure and exhibits high binding affinity for Cas12a by occupying its substrate DNA-binding site, thereby interfering with Cas12a-DNA interactions. Inspired by these findings, we explored whether this inhibitory aptamer could serve as a signal transducer for rapid *E. coli* detection. Here, we present an allosteric probe-regulated Cas12a/crRNA-mediated aptasensor for sensitive and reproducible bacterial detection. The design involves an engineered allosteric probe containing both a target recognition sequence and the Cas12a inhibitory aptamer. This probe exhibits target-dependent conformational switching: in the absence of the target, it adopts an active flap structure in which the core nucleotides bind to the Cas12a−crRNA complex, blocking its interaction with substrate dsDNA and reducing catalytic activity; upon target binding, it shifts to an inactive conformation, releasing the Cas12a/crRNA complex and restoring its dsDNA-binding capacity, thereby activating the tunable trans-cleavage activity of Cas12a toward the ssDNA reporter. This approach offers several advantages, including high sensitivity, operational simplicity, and excellent specificity. When applied to detect *E. coli* in various matrices, the aptasensor demonstrated high selectivity with minimal interference from other bacterial species.

## Experimental

### Reagents and Materials

The oligonucleotides were commercially synthesized by Sangon Biotech Co., Ltd., (China; see Table S1 for sequences). The aptamer for *E. coli* recognition was obtained from previous studies [21]. All bacterial strains used in this study were obtained from the strain repository of Chongqing Center for Disease Control and Prevention. The Cas12a protein and corresponding reaction buffer (10× Cas12a Reaction Buffer) were purchased from New England Biolabs (USA).

### Bacterial Cultures

In this study, *E. coli* was employed as a reference strain to assess the detection capability of the aptasensor and optimize the biosensor’s operational parameters. A pre-inoculum was prepared by culturing *E. coli* in 5 ml of Luria-Bertani (LB) broth (Sigma, USA) at 37°C with shaking at 220 rpm overnight. Subsequently, 100 μl of the pre-inoculum was transferred into 10 ml of fresh LB broth and incubated under the same conditions for 2 h to reach the exponential growth phase. The bacterial cells were harvested by centrifugation at 4,000 rpm for 15 min and resuspended in 1× PBS. Viable cell counts were determined through serial dilution, plating on LB agar (Sigma), and overnight incubation at 37°C. Colony-forming units per milliliter (CFU/ml) were calculated after enumeration. To evaluate the specificity, Pseudomonas aeruginosa (ATCC 15422) and *Staphylococcus aureus* (ATCC 6538), representing Gram-negative and Gram-positive bacteria, respectively, were used as control strains.

### Detection Procedures

The detection of *E. coli* was performed in two sequential steps: target recognition and signal output. For target recognition, a 10 μl aliquot of *E. coli* solution at varying concentrations was incubated with 25 nM sensing molecule, 1 U/μl RNase inhibitors, and 1× NEBuffer 3.1 at 37°C for 15 min. Subsequently, 20 nM Cas12a and 10 nM crRNA were introduced, adjusting the total volume to 18 μl, followed by incubation at 37°C for an additional 10 min. In the signal output step, 25 nM dsDNA (500 nM) was added to the reaction mixture, bringing the final volume to 20 μl, and incubated at 37°C for 75 min. The resulting solution was then transferred to a black 384-well microplate, and fluorescence was measured using a microplate reader with an excitation wavelength of 645 nm.

## Results and Discussion

### The Working Principle of the Biosensor

**Fig. 1:**
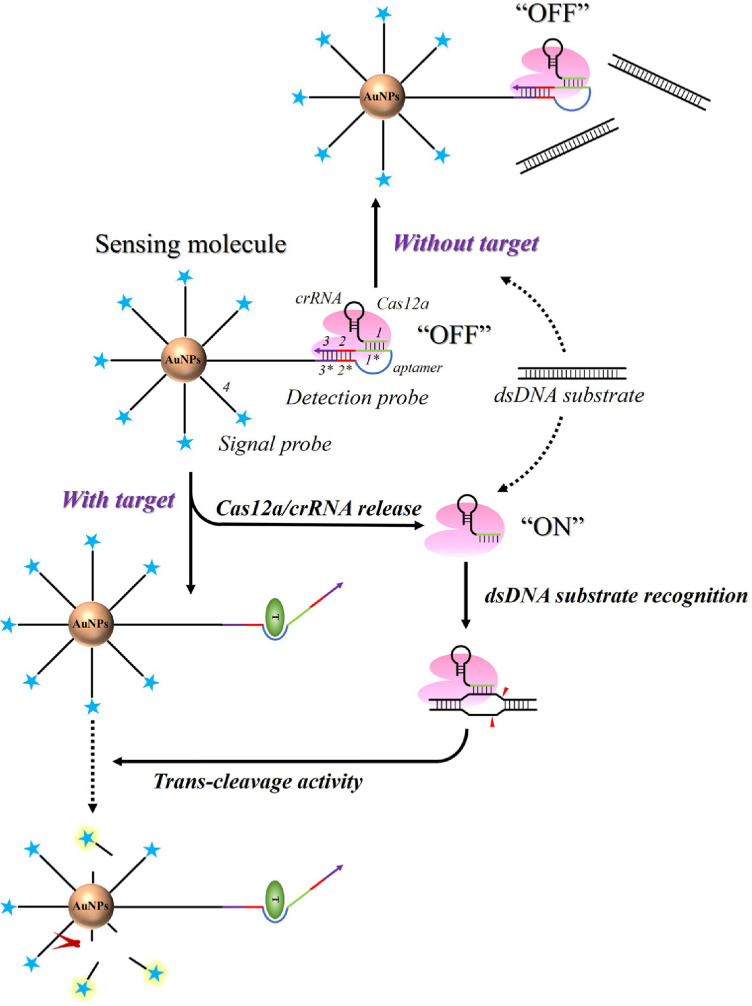
Working principle of the proposed method based on an allosteric probe controllable Cas12a/crRNA mediated signal amplification. T, Target bacteria (*E. coli*).

The proposed approach integrates two core elements: a detection molecule and a dsDNA substrate (Fig. 1). The sensing molecule is constructed by immobilizing the detection probe and signal probes on the surface of gold nanoparticles (AuNPs), whereas the dsDNA substrate activates the Cas12a/crRNA complex. The detection probe combines an allosteric probe with Cas12a/crRNA, maintaining the latter in an inactive (“OFF”) state. A Cy5-labeled “4” sequence (signal probe) is anchored to the AuNPs surface, leading to efficient fluorescence quenching by AuNPs. This method employs an allosteric detection probe that transduces target recognition into Cas12a's trans-cleavage activity. Structurally, the detection probe contains several functional domains: (i) an aptamer sequence for target binding, (ii) a “1” seed region for crRNA interaction, (iii) a “2” protospacer adjacent motif (PAM, 5'-NTT-3') for Cas12a binding, and (iv) complementary “3” and “3” sequences forming a stem to stabilize the hairpin conformation. The seed sequence “1” is strategically positioned near the PAM sequence “2”, mimicking substrate dsDNA and inhibiting Cas12a/crRNA activity. In the target’s absence, the detection probe occupies the substrate-binding site of Cas12a/crRNA, preventing recognition of substrate dsDNA and maintaining Cas12a in its inactive state, which preserves the intact signal probes. Upon target binding, the detection probe undergoes conformational changes that separate the seed sequence “1*” from the PAM sequence “2”, disrupting its interaction with Cas12a/crRNA. This restoration of Cas12a activity enables cleavage of both the substrate dsDNA and the signal probes, generating a quantifiable fluorescent signal proportional to the target concentration.

### Development of the Sensing Molecule and Evaluation of Approach Feasibility

**Fig. 2:**
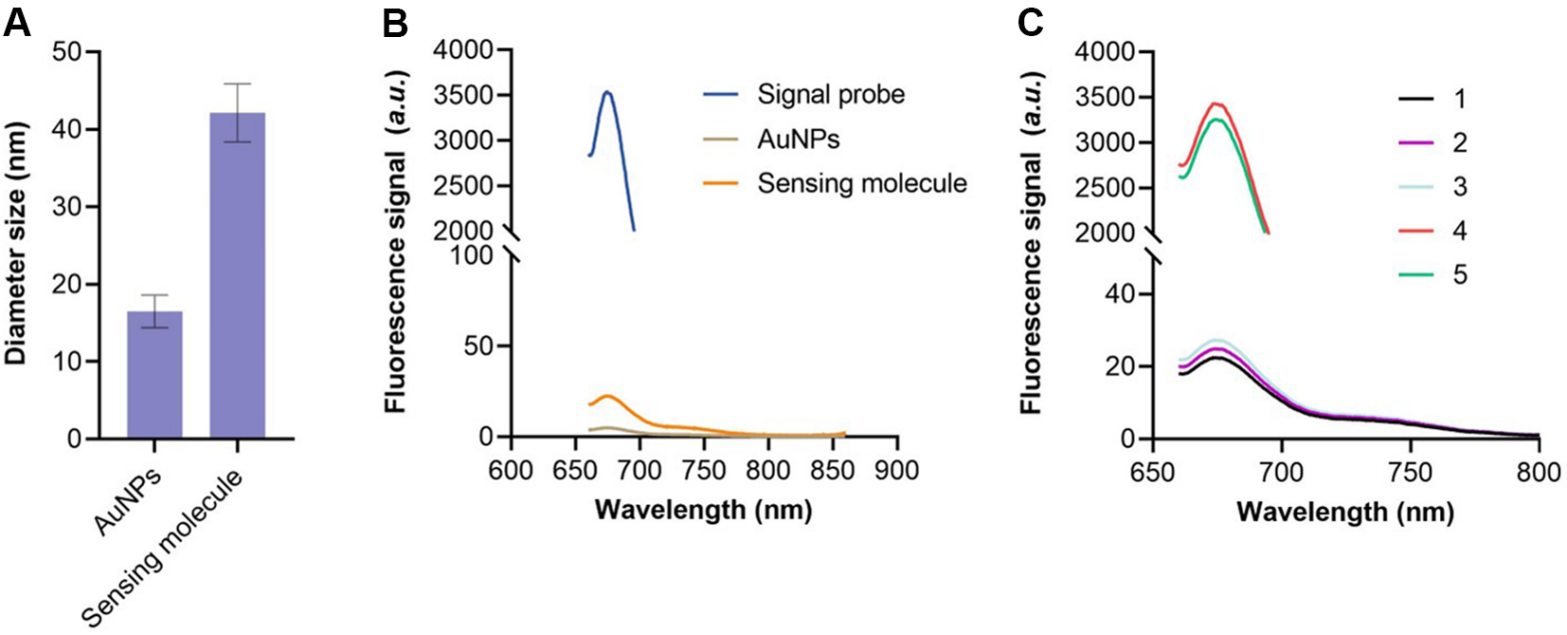
Feasibility of the biosensor for sensitive bacteria detection. (**A**) Diameter distribution of the AuNPs and sensing molecule. (**B**) Fluorescence signal of the signal probe before and after loading on the surface of AuNPs. (**C**) Fluorescence spectrum of the method when ssDNA substrate and target bacteria existed or not.

The conjugation of detection and signal probes to AuNPs plays a critical role in modulating the dsDNA substrate recognition capability of switch Cas12a/crRNA. The two probes were co-immobilized onto AuNPs *via* Au–S bonds to form a functional sensing complex. Dynamic light scattering (DLS) analysis indicated an increase in the average hydrodynamic diameter from 16.5 nm to 42.1 nm (Fig. 2A), while fluorescence measurements of Cy5-labeled signal probes before and after adsorption onto AuNPs revealed a quenching efficiency of 95.12%(Fig. 2B). These results confirm the successful assembly of the sensing complex.

As shown in Fig. 2C, the sensing complex exhibited minimal fluorescence, indicating efficient quenching of the Cy5 signal by AuNPs (line 1). Upon target bacteria binding, the hairpin structure of the detection probe underwent a conformational change, activating Cas12a/crRNA and triggering signal probe cleavage, thereby restoring fluorescence (line 4). In contrast, the absence of target bacteria or key components resulted in no detectable fluorescence (lines 2 and 3). Notably, the fluorescence signal was 34.12% lower when detection probes were not immobilized on AuNPs (line 5). Comparative analysis of fluorescence intensities between lines 5 and 4 demonstrated that probe immobilization on AuNPs significantly improves signal generation efficiency, likely enhancing detection sensitivity.

Additionally, we added an experiment to test the specificity of aptamer in recognizing target bacteria. The results in Fig. S1 showed the only the target aptamer induced a significantly elevated fluorescence signal. The above results indicated the high specificity of the method.

### Optimization of Experimental Parameters for Better Detection Performance

**Fig. 3:**
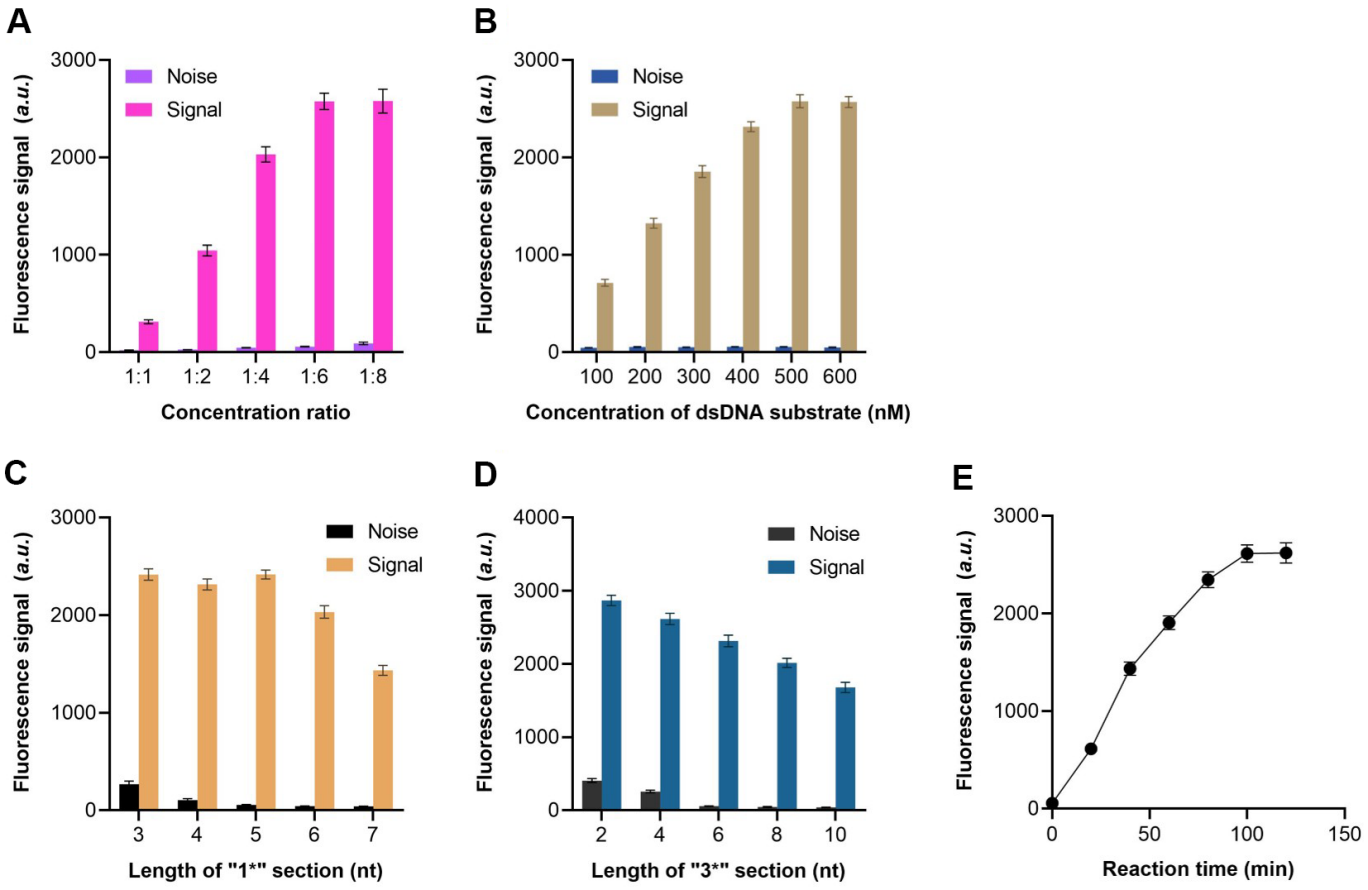
Optimization of experimental parameters. Fluorescence signals of the method when detecting target bacteria with different concentration ratio of detection probe and signal probe (**A**) concentration of dsDNA substrate (**B**) length of “1*” section (**C**) length of “3” section (**D**) and reaction time (**E**).

The sensing performance of this method is significantly influenced by several critical reaction parameters, such as the ratio of detection probe to signal probe on AuNPs and the concentration of the ssDNA substrate, all of which require systematic optimization. Initially, the ratio of detection probe to signal probe was optimized by evaluating sensing efficiency through comparative analysis of fluorescence intensity in the presence and absence of target microorganisms. The optimal ratio was determined to be 1:6 (Fig. 3A). Subsequently, varying concentrations of dsDNA substrate were introduced into the system, both with and without target bacteria, to obtain corresponding fluorescence spectra. The greatest fluorescence intensity changes were observed at 500 nM (Fig. 3B), confirming this as the optimal concentration.

Further investigation focused on the seed length and stem length of HPATP, as these parameters critically influence crRNA spacer interaction and detection probe structural stability, respectively. A series of detection probes with varying seed lengths (4, 5, 6, and 7 nt) and stem lengths were synthesized, and their fluorescence signals were independently analyzed (Fig. 3C and 3D). As expected, background signals decreased with increasing seed length from 4 to 7 nt, suggesting enhanced interaction between the detection probe and the Cas12a/crRNA complex. However, at 7 nt, the extended seed sequence formed a stable association with the crRNA spacer, significantly reducing Cas12a’s collateral cleavage activity, while modifications in hairpin structure did not affect seed-crRNA binding. Consequently, the 5 nt seed length demonstrated the highest signal-to-noise ratio (S/N). Similarly, the optimal stem length was determined to be 6 nt. Additionally, reaction time was evaluated, revealing a steady increase in S/N from 0 to 100 min before reaching equilibrium (Fig. 3E).

### Application of the Biosensor for *E. coli* Detection

**Fig. 4:**
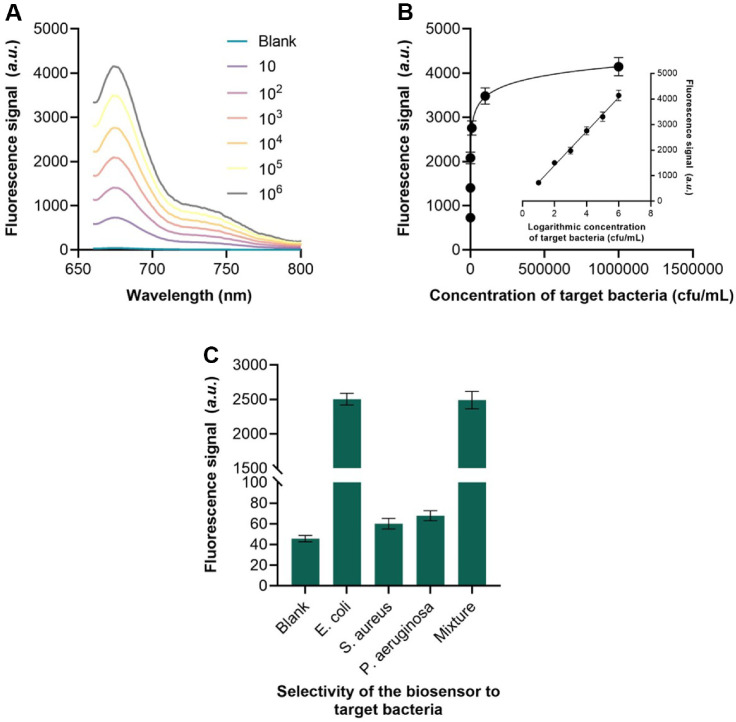
Analytical performance of the method. (**A**) Fluorescence spectrum of the method for different concentrations of *E. coli* detection. (**B**) Fitting curve of the fluorescence intensities and concentrations of target bacteria. (**C**) Fluorescence intensity of the method when detecting *E. coli* and interfering bacteria.

The analytical performance of the aptasensor was evaluated by measuring *E. coli* concentrations ranging from 10 to 10^6^ CFU/ml. As shown in Fig. 4A, the fluorescence intensity increased proportionally with each logarithmic increase in bacterial concentration. A linear relationship was observed between the fluorescence signal at 655 nm and *E. coli* concentrations across the dynamic range of 10–10^6^ CFU/ml (Fig. 4B). The calibration curve followed the equation F = 665.1 × lgC + 75.70 (R² = 0.984), with a detection limit of 4.6 CFU/ml, calculated as three times the standard deviation of the blank signal. The exceptionally low detection limit of 4.6 CFU/ml significantly enhances the sensitivity of this *E. coli* detection method, enabling reliable identification of the pathogen even at very early stages of contamination or infection when bacterial loads are minimal. To evaluate selectivity, the aptasensor was tested against potential interfering bacteria, including P. aeruginosa and S. aureus, which often coexist with *E. coli* in clinical samples. The results (Fig. 4C) demonstrated high specificity for *E. coli*, with negligible cross-reactivity toward *P. aeruginosa* or *S. aureus*, confirming the selective binding capability of the aptamer.

### *E. coli* Detection in Real Samples

To evaluate the applicability of this method to real-world samples, commercial serum samples were spiked with varying concentrations of *E. coli*. As demonstrated in Table 1, the proposed detection method achieved satisfactory recovery rates ranging from 97% to 105.4% for *E. coli* in these samples, with relative standard deviation (RSD) values between 4.54% and 5.12%, indicating good reproducibility. These results suggest the method’s potential for reliable *E. coli* detection in complex biological matrices.

**Fig. 5:**
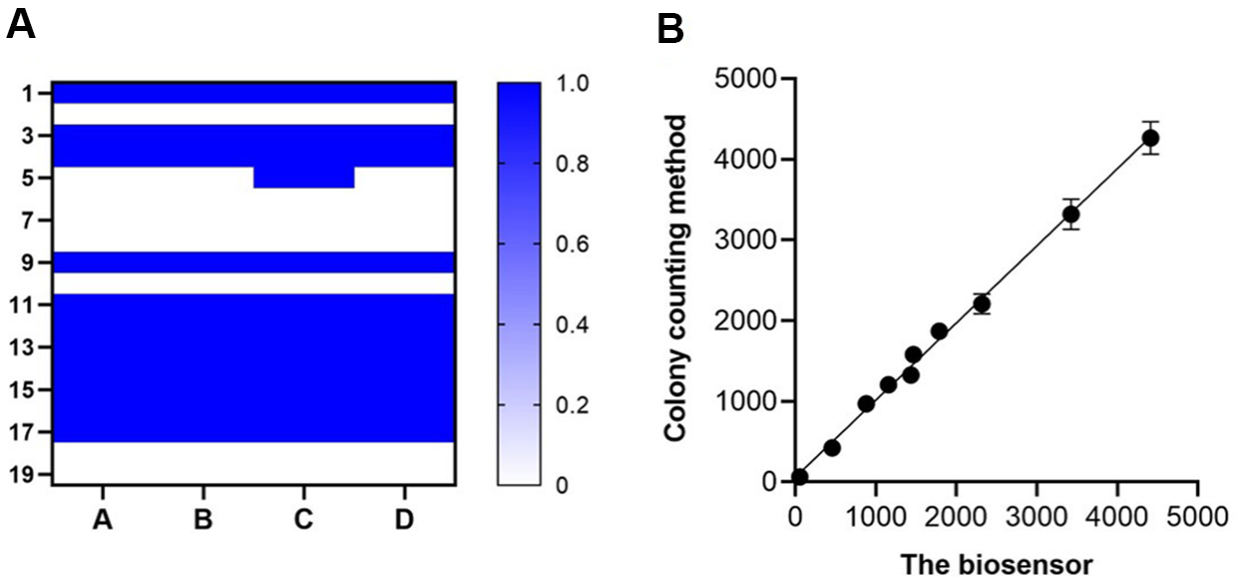
Application potential of the method. (**A**) Heat map of the detection results of the 20 samples by the method, colorimetric method and traditional colony counting method. (**A**) Added bacteria concentration; (**B**) the biosensor; (**C**) colorimetric method; (**D**) colony counting method. (**B**) Correlation between the calculated bacteria concentrations by the biosensor and colony counting method.

To further assess detection performance, 20 samples containing either the target bacterial strain or interfering strains were analyzed using the proposed technique, colorimetric testing, and conventional colony counting. Both the proposed method and colony counting identified *E. coli* in 12 clinical samples (Fig. 5A). Additionally, the detection results for MRSA using the proposed method were consistent with those obtained *via* conventional methods. A strong correlation (R² = 0.9987) was observed between bacterial concentrations measured by the proposed method and colony counting (Fig. 5B), demonstrating high agreement between the two approaches. These findings support the potential clinical utility of the proposed detection method.

## Conclusion

Herein, we present a novel strategy involving a structure-switchable detection mechanism that integrates a target recognition sequence with the inhibitory aptamer of the Cas12a system to precisely regulate CRISPR/Cas12a activity for bacterial detection. This approach significantly improves substrate cleavage efficiency through co-immobilization of the substrate and detection probes on gold nanoparticle (AuNP) surfaces. The designed system exhibits several key advantages: (1) high target recognition specificity mediated by both the aptamer and the Cas12a/crRNA complex; (2) superior signal amplification capability with a remarkably low detection limit of 4.6 cfu/mL, achieved through trans-cleavage activity of Cas12a and the synergistic effect of probe immobilization on AuNPs [22]. Additionally, this platform holds potential for multiplex analyte detection by employing specific inhibitors targeting distinct cleavage activities of Cas proteins. This study primarily aims to demonstrate the utility of CRISPR/Cas inhibitory aptamers as effective regulatory elements in biosensing systems, particularly for postoperative infection monitoring. Compared with traditional CRISPR-based bacteria detection methods and PCR based method, the proposed method can identify target bacteria without the extraction of genomic material, simplifying the experimental procedures [23, 24]. We anticipate that this strategy will inspire further development of advanced aptamer-based CRISPR/Cas biosensors.

However, the high specificity of the method for *E. coli*, conferred by the carefully selected aptamer probes, inherently restricts its direct utility for detecting other bacterial species; applying this specific assay to different bacterial targets would necessitate the design and validation of entirely new, organism-specific aptamer probes. Therefore, future work will focus on further optimization strategies, such as developing multiplexed aptamer panels or engineering broader-spectrum recognition elements, to transform this platform into a versatile tool capable of simultaneously detecting multiple pathogenic bacteria.

## Supplemental Materials

Supplementary data for this paper are available on-line only at http://jmb.or.kr.



## Figures and Tables

**Table 1:** Recovery test of the assay.

Samples	Added concentration (CFU/ml)	Obtained concentration (CFU/ml)	Recovery rate
1	500	485	97
2	1000	1054	105.4
3	5000	5103	102.6
