# Supplementary material for: Simple and Sensitive Escherichia coli Analysis via Allosteric Probe Controllable Switch Cas12a/crRNA Complex Mediated Strategy
Source: J Microbiol Biotechnol. 2025 Aug 28;35:e2506010. doi: 10.4014/jmb.2506.06010 (PMC12409431; doi:10.4014/jmb.2506.06010)
Supplement: Supplementary file 1 [file jmb-35-e2506010-supple.pdf]

J. Microbiol. Biotechnol. 2025. 35: e2506010

https://doi.org/10.4014/jmb.2506.06010

Supplementary Table and Figure

Table S1. **Sequence information for oligonucleotides was used in this study.**

Title	Sequences (5' to 3')
Detection probe	CGT TTG ACC TGG GGG AGC ATT GCG GAG GAA GGT CTG AGC AAA CG
Signal probe	FAM-TTA TTT
DP-4 seed	CGTTTGACCTGGGGGAGCATTGCGGAGGAAGGTTGAGCAAACG
DP-5 seed	CGTTTGACCTGGGGGAGCATTGCGGAGGAAGGTCTGAGCAAACG
DP-6 seed	CGTTTGACCTGGGGGAGCATTGCGGAGGAAGGTGCTGAGCAAACG
DP-7 seed	CGTTTGACCTGGGGGAGCATTGCGGAGGAAGGTCGCTGAGCAAACG
DP-4	TTTGACCTGGGGGAGCATTGCGGAGGAAGGTCTGAGCAAA
DP-6	CGTTTGACCTGGGGGAGCATTGCGGAGGAAGGTCTGAGCAAACG
DP-8	GTCGTTTGACCTGGGGGAGCATTGCGGAGGAAGGTCTGAGCAAACGAC
DP-10	CAGTCGTTTGACCTGGGGGAGCATTGCGGAGGAAGGTCTGAGCAAACGACTG

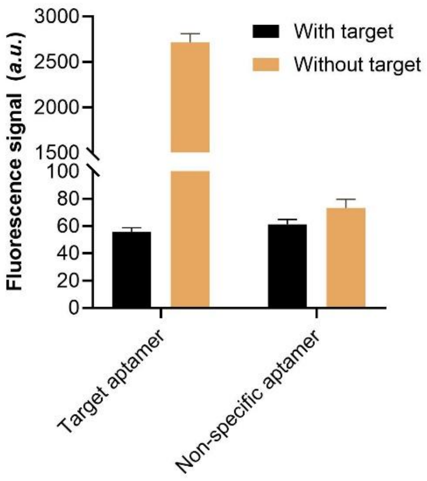

Fig. S1. Fluorescence intensity of the method when using target aptamer or non-specific aptamer.
